# Case Report: A case of carcinoma *in situ* of the bladder misdiagnosed as “chronic prostatitis” for a long time

**DOI:** 10.3389/fonc.2025.1632624

**Published:** 2025-09-11

**Authors:** Chongbin Li, Guiyun Zhu, Jianzhen Liu, Wei Li

**Affiliations:** ^1^ Department of Urology, The Second Hospital of Hebei Medical University, Shijiazhuang, China; ^2^ Department of Urology, Hebei Chest Hospital, Shijiazhuang, China; ^3^ Department of Pathology, Hebei Chest Hospital, Shijiazhuang, China

**Keywords:** bladder, chronic prostatitis, carcinoma *in situ* (CIS), patholgoy, cytology, biopsy

## Abstract

**Background:**

Carcinoma *in situ* of the bladder (CIS) has no specific clinical symptoms and is easily confused with inflammatory lesions of the bladder and urethra. It is usually not considered as the primary diagnosis and requires cystoscopic biopsy to confirm the diagnosis.

**Case presentation:**

A 44-year-old male patient was diagnosed with chronic prostatitis due to “intermittent urinary frequency, dysuria, and urethral dribbling after defecation” and was treated with intermittent anti-infective and symptomatic therapy for 2 years, with symptoms recurring after discontinuing the medication. This patient underwent urine exfoliative cytology, which revealed exfoliated cancer cells, and proceeded to undergo cystoscopic biopsy, which did not reveal a pathologic basis for the cancer. The diagnosis of CIS was finally confirmed only after a diagnostic transurethral bladder mucosal electrodesiccation and pathologic examination. Bacillus Calmette-Guerin (BCG) bladder instillation was then performed for up to one and a half years, and the patient recovered.

**Conclusion:**

Bladder carcinoma *in situ* can present with urinary irritation symptoms similar to those of prostatitis and is therefore easily misdiagnosed as chronic prostatitis. It is recommended for patients with recurrent urinary frequency and dysuria as the main symptoms to undergo urine exfoliative cytology routinely if bladder tumor cannot be ruled out, and cystoscopy and biopsy should be performed if necessary to rule out CIS.

## 1 Introduction

CIS, also known as “flat carcinoma”, is a high-grade, non-invasive bladder cancer that is generally poorly differentiated, has a high risk of basal infiltration, and is a highly malignant tumor. Its clinical symptoms are similar to those of inflammatory diseases of the bladder and urethra, manifested by frequent urination, painful urination, and, occasionally, hematuria. In cystoscopy, CIS is also easily confused with inflammatory bladder disease, and biopsy is required to confirm the diagnosis. In addition, CIS is often multifocal and can also be seen in the upper urinary tract or prostatic urethra. Clinically, CIS is generally not the preferred diagnosis and is mostly considered after inflammatory diseases of the bladder and urethra have been ruled out, making it easy to miss and misdiagnose.

## 2 Case presentation

### 2.1 Initial presentation and misdiagnosis

A 44-year-old middle-aged man was diagnosed with chronic prostatitis in a provincial general hospital due to “intermittent frequent urination, painful urination, and urethral dribbling after defecation for 2 years” and was intermittently treated with antibiotics (cephalosporin) and proprietary Chinese medicines (Relinqing Keli and LongQing JiaoNang) for 2 years. The medication was discontinued after the symptoms improved, and the symptoms have recurred four times so far (**Table 1**).

**Table 1:** Summary of diagnosis and treatment process.

Timeline	Diagnosis	Examination methods and results	Treatment	Therapeutic efficacy
In the past 2 years	Chronic prostatitis	The results of color Doppler ultrasound examination of the urinary system, routine prostate fluid testing, and urine analysis were not available, but the diagnosis of chronic prostatitis was made based on the symptoms (especially “urethral dribbling”).	Antibiotics and proprietary Chinese medicines	Not yet cured, symptoms recur repeatedly.
Since this visit	1. Urinary tract infection 2. Chronic prostatitis?	The results of urinary system CT scan (plain and contrast-enhanced), PPD test, T-SPOT.TB, routine urine bacterial culture, *Mycobacterium tuberculosis* culture, voiding diary, and other relevant tests indicated mild urinary tract infection or inflammation, ruling out urological tuberculosis.	Antibiotic and symptomatic treatment	Symptoms improved significantly.
	CIS	Cancer cells were detected in the urine cytology test.	None	
		Cystoscopy biopsy: Hyperplasia of the urinary tract epithelium with focal moderate to severe atypical hyperplasia	None	
		Diagnostic transurethral resection of the bladder mucosa, with postoperative pathological results confirming bladder carcinoma *in situ*.	BCG bladder instillation therapy for 18 months	CIS was cured. After 7 years of follow-up, there was no recurrence, but there was still mild urinary frequency and mild urinary discomfort when drinking less water.

### 2.2 Evaluation at our hospital

Before visiting our urology department, the patient had suffered from allergic rhinitis for 4 years, felt physically and mentally exhausted in the current 2 years, and had a history of high-risk sexual intercourse but not smoking. The patient had been repeatedly examined and treated for approximately 2 years in other hospitals before coming to our hospital’s urology department. The prostate fluid routine examination showed lecithin microsomes (++) and leukocytes 2–3/HPF. Urological ultrasound showed calcified spots in the right renal parenchyma, enlarged volume of the prostate gland, and no space-occupying lesions in the left kidney and bladder. Urine routine examination showed a leukocyte level of 26.4/μL (reference range: 0–25/μL) and a PSEP (prostatic exosomal protein) level of 1.28 ng/mL (reference range: 0–1.2 ng/mL), which indicate that the results were mild abnormal. After the patient was admitted to the Department of Urology at our hospital, we performed relevant tests to evaluate the condition, clarify the diagnosis, and guide the treatment (**Table 1**).

### 2.3 Diagnostic procedures leading to confirmation and the treatment accordingly

Firstly, the urological plain and enhanced computed tomography (CT) scan showed no abnormalities in the kidney, ureter, and bladder; no enlarged lymph nodes or free fluid was seen; and no neoplastic occupations or typical lesions of other diseases, such as tuberculosis, were detected in the urinary system. The urination diary showed frequent urination (18–30 times/day), accompanied by painful urination, urgency, abdominal pain, and discomfort in the anal area, which could be alleviated by lying on the side in the bladder area, accompanied by significant anxiety and sleep disturbances (insomnia and awakening from nightmares). NIH-CPSI (National Institutes of Health-chronic prostatitis symptom index) showed that pain score was 15, urinary symptoms score was 8, and the score of impact on quality of life was 11 (**Table 1**).

Secondly, to rule out bladder tuberculosis, we also performed the following tests. Chest x-ray showed no abnormality, suggesting that the patient did not suffer from lung tuberculosis. The PPD test result was strongly positive, suggesting active *Mycobacterium tuberculosis* infection. The result of T-SPOT.TB showed 8 SFCs/2.5×10^5^ peripheral blood mononuclear cells (PBMCs), suggesting a positive (+) finding. PCR TB-DNA and PCR NTM-DNA tests (conducted six times) and consecutive acid-fast staining in urinary sediment (conducted three times) showed negative (−) results. Tuberculosis antibody tests (38 and 16 kDa) also showed negative (−) results, while LAM had a positive (+) result. Consecutive tuberculosis cultures of the urine (three times) did not show growth of *M. tuberculosis*. After consultation with tuberculosis experts, diagnostic anti-tuberculosis drug therapy was recommended for 1 month to observe the change in condition. However, the patient refused to be treated with anti-tuberculosis drugs until the cause of the disease was clearly determined (**Table 1**).

Thirdly, as the patient had a history of high-risk sex, the following tests were performed in order to rule out sexually transmitted diseases. HbsAg, HBsAb, HBeAg, HBeAb, HBcAb, HIV, TP, and HCV tests showed negative (−) results. Moreover, no abnormalities were detected in routine blood, urine, and fecal tests; the fecal occult blood test; and the erythrocyte sedimentation rate (ESR) test. *Arcanobacterium haemolyticum* was observed in urine general bacterial culture, which was resistant to ciprofloxacin, clindamycin, ampicillin, benzathine, azithromycin, and erythromycin, and susceptible to antibiotics such as levofloxacin, cefuroxime, amikacin, and gentamicin (K-B method). Urinary mycoplasma culture showed negative (−) results. The urine two-cup test showed that urine leukocytes and general bacterial cultures before and after prostate massage were all negative (−). Thus, our diagnosis is urinary tract infection and suspected chronic prostatitis. Based on the results of the drug sensitivity test, the patient received anti-infective treatment (levofloxacin injection, 0.5 g IV QD) and symptomatic treatment [tamsulosin hydrochloride extended-release capsule, 0.2 mg PO QD; LongBiShu JiaoNang (KeDi), 0.9 g PO BID; Prostat tablets, 1 tablet PO BID; Prostant, 1 pill, PR QN; and phloroglucinol for injection, 80 mg IV QD]. After 7 days of treatment according to the above regimen, the symptoms of frequent and painful urination were significantly relieved but continued to persist. Moreover, since the patient came to our hospital, no urethral dribbling has been observed (**Table 1**).

**Figure 1:**
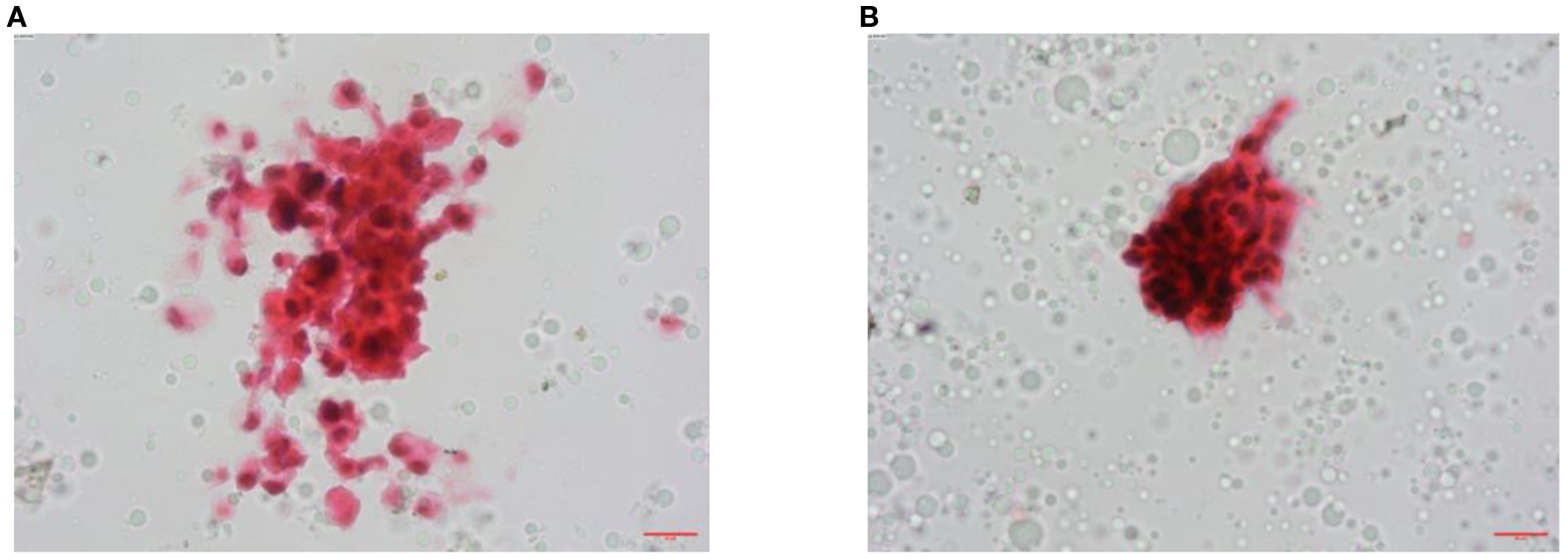
Pathological examination images. **(A)** Urine exfoliative cytology found suspicious cancer cells, HE staining ×400 (no. A1804386). **(B)** Urine exfoliative cytology found suspicious cancer cells, HE staining ×400 (no. A1804407).

Fourthly, in order to rule out urinary tumors such as CIS, we performed three consecutive urine exfoliative cytology examinations on the patient, and the surprising result was that suspicious tumor cells (+/−) were found in two of them (**Figures 1A, B**, pathology numbers: A1804407 and A1804386). In order to further define or exclude a urinary tract tumor, the patient underwent cystoscopy, which revealed a large area of mucosal congestion and edema and diffuse ulceration in the right posterior wall of the bladder. Bladder biopsy results (pathology number: A120017996) showed fibrovasculitis polyp proliferation and focal uroepithelial atypia. However, the pathologic findings failed to provide practical value for clinical treatment. Subsequently, consultation results from the pathology section of the Chinese People’s Liberation Army General Hospital showed inflammatory granulation tissue and hyperplastic uroepithelium with focal moderate-to-severe atypical hyperplasia. The patient was recommended to undergo diagnostic electrosurgery and pathology examination to clarify the diagnosis (**Table 1**).

Lastly, the patient underwent transurethral cystic mucosal diagnostic electrodesiccation at Peking Union Medical College Hospital, and the postoperative pathologic results finally confirmed a diagnosis of CIS. Afterwards, the patient underwent 18 months of Bacillus Calmette-Guerin (BCG) bladder instillation at Peking University First Hospital (Bisacodyl 120 mg, 6 times of inductive instillation once a week, 3 times of intensive instillation once every 2 weeks, and 16 times of maintenance instillation once a month) (**Table 1**).

### 2.4 Follow-up

Cystoscopy was performed every 3 months during the period of BCG bladder instillation and every 6 months for 3 years after the end of the treatment. All biopsies revealed chronic interstitial cystitis; cystoscopy was performed annually thereafter. To date, the patient has been followed up for 7 years and there has been no recurrence of CIS. At present, the patient continues to experience mild urinary frequency and mild urinary discomfort with little water intake, with approximately 200 mL of urine each time; everything else is normal.

## 3 Discussion

In this case of CIS, the following diagnostic difficulties were encountered:

First, the diagnosis of “chronic prostatitis” was made due to the very similar and confusing clinical symptoms of “frequent urination, painful urination, and urethral dribbling after defecation”, which proved to be incorrect. These are typical symptoms of prostatitis, especially the phenomenon of “urethral drip” after defecation. Moreover, chronic prostatitis is prone to recurrence if the cause of the disease is not addressed. Therefore, this is the main reason for the 2-year misdiagnosis. However, when the CIS invades the urethra and prostate (1, 5, 6), it may also stimulate the prostate to secrete prostatic fluid and cause the urethral “dribbling” phenomenon. It has been reported that 15% of CIS can undergo pagetoid-like invasion, in which cancer cells grow beneath the normal urinary tract epithelium and invade the prostatic urethra, leading to *in situ* carcinoma of the prostatic urethra or infiltration of the prostatic stroma (2). Although the patient was examined for prostatitis, including the urine “two-cup test” (1), the results did not support the diagnosis of prostatitis, probably because prostatitis had already been cured by the patient’s long-term and repeated anti-infective treatments, and the CIS was concurrent or subsequent to the prostatitis. Therefore, clinicians should be quick to identify and exclude CIS in patients who were first diagnosed with prostatitis or other inflammatory diseases in the urinary system due to urinary frequency and dysuria, especially for those whose standard anti-infective treatment proved to be ineffective.

Secondly, cancer *in situ* of the bladder is a type of highly malignant bladder tumor, 40%–83% of which can infiltrate the muscle layer of the bladder and develop distant metastasis at an early stage, endangering patients’ lives. Its early clinical manifestations also show frequent and painful urination, which are very similar to the clinical symptoms of urinary tract infection and prostatitis. For example, in 1985, the Mayo Clinic reports that 80% of CIS patients have urinary tract irritation symptoms (Zincke et al, 1985). Moreover, early CIS has no special abnormal manifestations on ultrasound and CT imaging. The sensitivity of urine exfoliative cytology is highly related to the tumor grade, with a low positive rate of only 16% for low-grade urinary tumors. However, because CIS is a type of high-grade malignant tumor and owing to the disruption of the function of intercellular adhesion molecules, cancer cells still have a high rate of positivity in urinary exfoliative cytology, with a high degree of sensitivity (84%) and a specificity of more than 90% [1~2]. Therefore, we recommend routine urine exfoliative cytology in cases with such symptoms and where bladder malignancy cannot be excluded.

Thirdly, CIS lesions are composed of severely dysplastic urothelial cells, which exhibit disorganized histological changes characteristic of highly malignant tumors under microscopic examination. However, because of the loss of cellular adhesion, partial or complete mucosal detachment may sometimes complicate pathological interpretation, leading to reports describing “abnormal hyperplasia” or “atypicality”. This ambiguity can confuse the diagnosis and make it difficult for clinicians to decide on the appropriate treatment. The pathological report in this case study also presents urologists with challenges in determining the appropriate course of further treatment. Most pathologists consider these cases to be benign; however, lesions with severe atypical hyperplasia or severe dysplasia are considered equivalent to CIS. Therefore, close communication between urologists and pathologists can minimize the risk of misunderstanding. In addition, since the prostatic urethra may be invaded by urothelial carcinoma, especially CIS, with pagetoid-like spread, urologists need to perform biopsies of the prostatic urethral epithelium with a normal appearance when evaluating patients with positive urinary cytology and a normal appearance on cystoscopy (2, 5, 6).

Lastly, urinary tuberculosis may also present with severe urinary frequency, pain, and cloudy urine, and in severe cases, “rice soup-like” pus urine may be seen (1, 2). However, urologic tuberculosis is more prominent on imaging, such as worm-eaten-like hypodense foci in the kidneys, and dilatation and wall thickening of the renal pelvis and ureter. Bladder tuberculosis rarely occurs in isolation and is only seen as a secondary infection after BCG bladder instillation therapy. In this case, although the patient was strongly positive for PPD (10×8 mm with blisters), and the T-SPOT.TB value was slightly higher at the critical level, imaging examinations did not reveal tuberculous lesions in the lungs, kidneys, and ureters, and consecutive tuberculosis cultures and TB-DNA were all negative, which basically ruled out urogenital tuberculosis (1–4). In addition, the results of the PPD test are also influenced by the proficiency level and experience of the interpreting physician. The diagnosis of urinary tuberculosis was also ruled out in this case, as the patient did not undergo anti-tuberculosis treatment to achieve clinical cure.

In conclusion, for patients with “urinary frequency and urinary pain” as the main symptoms, negative for urinary tract infection per urinalysis results, and without special abnormalities per imaging findings, clinicians should not be quick to arrive at a prostatitis diagnosis, especially since a diagnosis of CIS is a possibility. Urine exfoliative cytology can be used as a routine screening tool, and cystoscopy and biopsy should be performed if necessary to rule out CIS.

## Data Availability

The original contributions presented in the study are included in the article/supplementary material. Further inquiries can be directed to the corresponding authors.
